# Correlated transitions in TKE and mass distributions of fission fragments described by 4-D Langevin equation

**DOI:** 10.1038/s41598-018-37993-7

**Published:** 2019-02-06

**Authors:** Mark Dennis Usang, Fedir A. Ivanyuk, Chikako Ishizuka, Satoshi Chiba

**Affiliations:** 1 Laboratory for Advanced Nuclear Energy, Institute of Innovative Research, Tokyo Institute of Technology, Tokyo, 152-8550 Japan; 2 Reactor Technology Center, Technical Support Division, Malaysia Nuclear Agency, Bangi, 43000 Kajang, Malaysia; 3 Nuclear Theory Department, Institute for Nuclear Research, Prospect Nauki 47, 03028 Kiev, Ukraine; 4 Theoretical Division, National Astronomical Observatory of Japan, Mitaka, Tokyo 181-0015 Japan

## Abstract

We have decomposed to symmetric and asymmetric modes the mass-TKE fission fragment distributions calculated by 4-dimensional Langevin approach and observed how the dominant fission mode and symmetric mode change as functions of ${Z}^{2}/\sqrt[3]{A}$ of the fissioning system in the actinides and trans-actinide region. As a result, we found that the symmetric mode makes a sudden transition from super-long to super short fission mode around ^254^Es. The dominant fission modes on the other hand, are persistently asymmetric except for ^258^Fm, ^259^Fm and ^260^Md when the dominant fission mode suddenly becomes symmetric although it returns to the asymmetric mode around ^256^No. These correlated “twin transitions” have been known empirically by Darleane Hoffman and her group back in 1989, but for the first time we have given a clear explanation in terms of a dynamical model of nuclear fission. More specifically, since we kept the shape model parameters unchanged over the entire mass region, we conclude that the correlated twin transition emerge naturally from the dynamics in 4-D potential energy surface.

## Introduction

The study of fission by Langevin equation in recent years has had some considerable success^1–10^, especially in unraveling the physics involved in the fission process. Recently, we have introduced the microscopic mass and friction tensors to improve the calculations of 3-D Langevin equation^7^ instead of the usual macroscopic mass and friction tensors^11–13^. With it, we see the average total kinetic energy 〈TKE〉 decreasing with larger excitation energy *E*_*x*_ and the influence of pairing at smaller *E*_*x*_^3^. There are some deficiencies with the 3-D Langevin model because we were unable to observe the expected transition from double peak fission yield of ^256^Fm to the single peak fission yield ^258^Fm, and the TKE as a function fragment mass TKE(*A*) are rather poor.

These two transitions, in terms of the anomalous changes in the fragment mass yield and TKE, are what we wish to explain. It was first observed experimentally by Hoffman *et al*.^14^ for ^258^Fm and were further corroborated by later experiments^15,16^. Hulet *et al*.^15^ even proposed that these transition occur as the fissioning nucleus splits into double magic fragments and the high TKE seen for ^258^Fm are due to the preference for super-short fission modes^17^. However, there was no clear explanation why the super-short fission modes are preferred at all instead of the super-long fission modes as was common for all the neighboring actinides.

Thus within the two-center shell model shape parameterization we^18^ took into account an additional degree of freedom by allowing the independent deformation of fission fragment tips, and this allowed us to improve TKE(*A*) even though we were only able to use it in conjunction with macroscopic transport coefficients. It seems that the improvements of TKE(*A*) is due to the strong relationship between the elongation of the fissioning system at the scission point and the TKE^19^. In 3-D Langevin approach, the dynamical variables are (*z*_0_/*R*_0_, *δ*, *α*) representing the elongation, fragment tip deformation and mass asymmetry respectively. We assume in 3-D Langevin approach the shape of the fission fragment tips of the left and right fragments to be the same (*δ* = *δ*_1_ = *δ*_2_). At present we are able to introduce an additional degree of freedom. Thus for 4-D Langevin approach, the dynamical coordinates are (*z*_0_/*R*_0_, *δ*_1_, *δ*_2_, *α*) to represent the elongation, right fragment tip shape, left fragment tip shape and mass asymmetry. Unfortunately, so far we are stuck with macroscopic transport coefficients when we are using 4-D Langevin equations.

We believe that the more commonly seen transition of fragment mass yield that occurred from ^256^Fm to ^258^Fm and its recovery at larger compound mass (or charge) are correlated to the anomalous transition of the TKE seen from the same nuclei. In the present work, we use the 4-D Langevin approach with macroscopic transport coefficients for studying the fragment mass and TKE distributions of various fissioning system from Uranium to Rutherfordium. Our aim is to look for the explanation of the transition from the double peak fragment yield of ^256^Fm to single peak fragment of ^258^Fm and at the same time, to explain the anomalous increase of 〈TKE〉 in the said transition.

## Results

**Figure 1:**
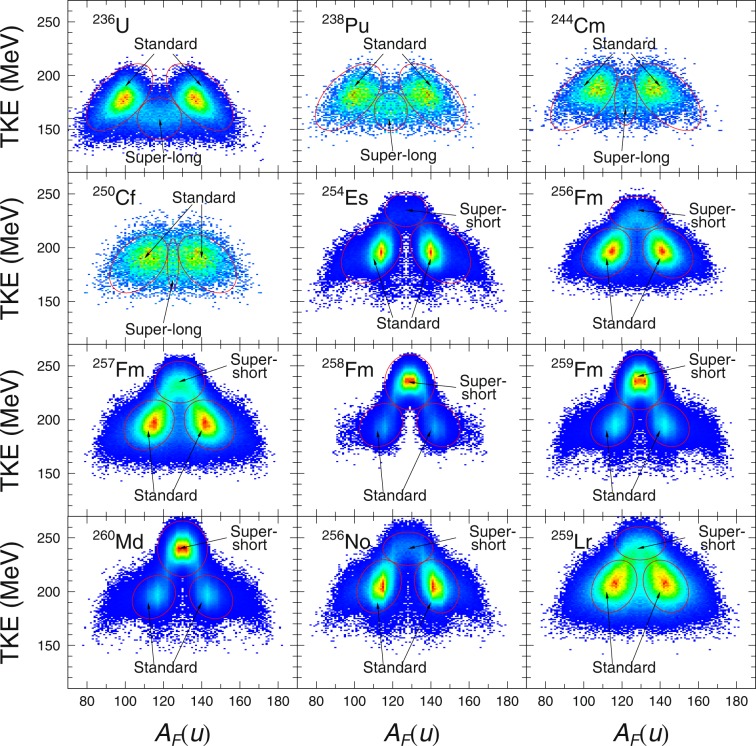
Fission fragment TKE profile from ^236^U to ^259^Lr as a function of fragment mass, *A*_*F*_(*u*). The color are linearly scaled from blue to red indicating the counts in arbitrary units.

The main observables from Langevin calculation are the fission fragment mass yield and TKE. Fission mass yield is calculated from the statistics of fragment mass given by the value of *α* at scission. Total Kinetic Energy is calculated from the sum of Coulomb repulsion and pre-scission kinetic energy. Brosa^17^ introduced several fission modes associating TKE with the shapes (or length) of the nuclei at scission. As the name of these fission modes indicates; the super-short fission modes, standard fission modes and super-long fission modes refers to the length of the nuclide with super-short fission modes being the shortest and super-long fission modes being the longest. In all the nuclei that we managed to calculate with 4-D Langevin, we are able to observe the ever present standard fission modes^17^ manifesting itself in the asymmetric TKE components. In the lighter fissioning system such as ^236^U, we could easily identify the presence of super-long fission modes (smaller TKE) in the symmetric components of the mass distributions. On the other hand, the heavier fissioning system such as ^258^Fm exhibits super-short fission modes (larger TKE) in the symmetric components. Snapshots of the TKE profile for a chosen nucleus representing the fissioning system from ^236^U all the way up to ^259^Lr can be observed in Fig. 1.

The average value of TKE is roughly proportional to *Z*_1_*Z*_2_/*R* and it also seems to increase with larger *Z*. The super-long fission mode slowly diminishes on the symmetric component in tandem with the increasing compound charge until at some point (^254^Es) it suddenly switches to super-short in the symmetric component. By drawing an ellipse over the fission modes that we have identified on each TKE profile and took the average TKE in the area spanned by the ellipse for the identified standard fission mode in the asymmetric component, we get TKE_asy_. We see TKE_asy_ increasing in tandem with the increase of *Z*. In a similar fashion we identify TKE_sym_ from the super-long or super-short fission modes. From Curium to Californium, it was not clear if any symmetric fission modes are present at all. For such cases, we simply took a narrow band around the symmetric mass as TKE_sym_ for the said nuclei. The symmetric component seems to have exclusively super-short fission modes for all heavier actinides onwards. The snapshot of the TKE profile from ^257^Fm to ^259^Lr in Fig. 1 illustrate these phenomena pretty well.

**Figure 2:**
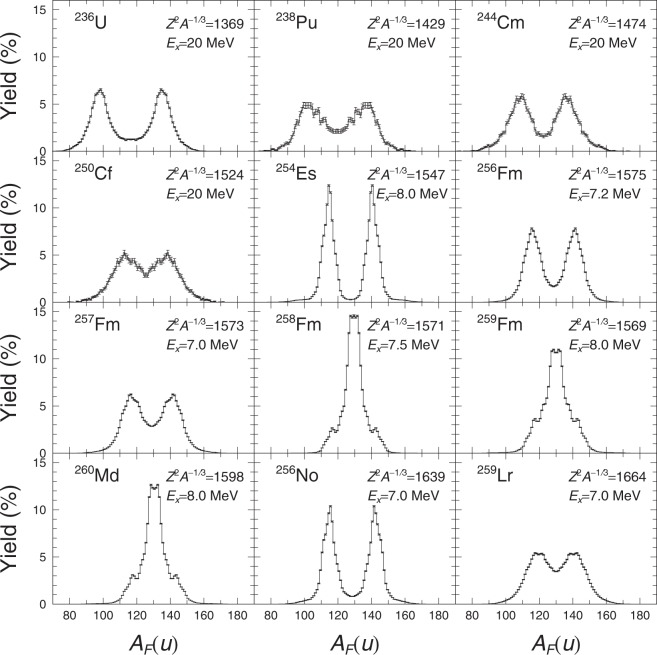
Fission fragment yield from ^236^U to ^259^Lr as a function of fragment mass *A*_*F*_(*u*). The yield are indicated in percentage.

In the case of ^258^Fm, ^259^Fm and ^260^Md, the fission fragments tend to have double magic configuration when they split symmetrically. Due to the preference for symmetric split the only symmetric fission modes available is super-short fission mode, hence it dominates the TKE profile. As a consequence, 〈TKE〉 are also pulled higher. Away from the double magic splits, we see that although super short fission modes are still the preferred symmetric fission mode, the asymmetric fission modes dominate instead. In the perspective of fission fragment mass yield, this meant that the usual two-peak fragment yield became single-peak for ^258^Fm, ^259^Fm and ^260^Md, and then switched back to double-peak fragment yield. In Fig. 2, we demonstrate the transition from the double peak ^256^Fm to the single peak ^258^Fm and the recovery of double peak fission fragment yield in ^259^Lr.

The presence of super-short fission modes for ^254^Es are independent from its excitation energy at 8 MeV. For example, at excitation energy of 20 MeV, ^254^Es TKE_asy_ component is around 195.74 MeV but its 〈TKE〉 is 197.19 MeV. The average TKE is pulled higher by the symmetric TKE components belonging to the super short fission modes. The super-short fission modes component averages around 225.82 MeV. Of course, the majority of the fission events split in an asymmetric manner, but the portion of events that does split symmetrically are due to super-short fission modes. The average TKE of the same nuclide for most fissioning systems, seem to vary with increasing excitation energy. It must be noted however that such variation are usually less than 5 MeV.

### Systematics

**Figure 3:**
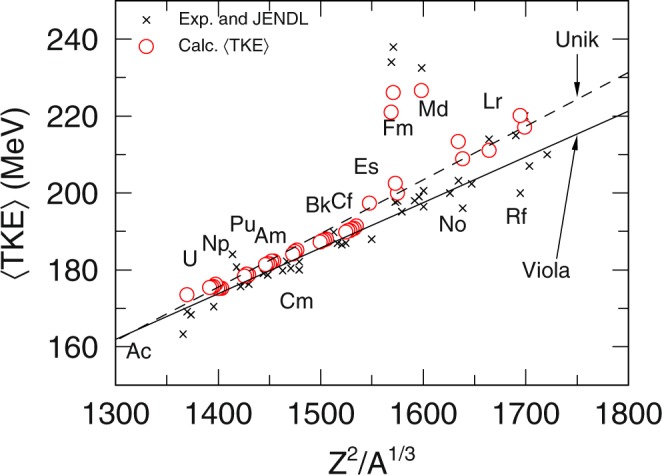
Experimental TKE^34,35^ and evaluated data^36^ denoted by (×) compared to calculated 〈TKE〉 () as a function of fissioning system.

**Figure 4:**
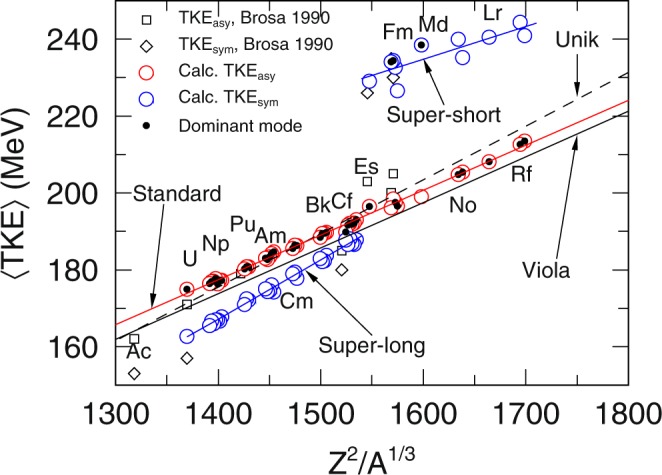
Calculated TKE_sym_ () and TKE_asy_ () and the associated trend given respectively by () and (). The dominant fission mode are marked by (•). Data is plotted as a function of fissioning system.

We can see from Fig. 3 that while all the other nuclei 〈TKE〉 seems to follow closely either the Viola systematics^20^ or the systematics of Unik (Double Energy Experiments)^21^, the three nuclei ^258^Fm, ^259^Fm and ^260^Md clearly deviates away from them. Both systematics are obtained by taking the linear fit of the TKE as a function of Coulomb repulsion from various fission experiments. If we take in the trends of the asymmetric TKE components (standard fission modes) from TKE_asy_, we get TKE_*STD*_ = 0.1168*Z*^2^*A*^−1/3^ + 13.9 MeV. It is most interesting to note how close the slope coefficient of Viola value is with the slope coefficient of TKE_*STD*_ across the various fissioning system. The results used in plotting both Figs 3 and 4 are tabulated in the Supplementary Information and it includes other notes on the particulars of the calculation.

With regards to the symmetric component, the super-long fission mode approaches that of the standard fission mode in Fig. 4 consistent to what we see in Fig. 1 with the disappearing super-long fission modes. Thus the TKE_sym_ for the range the fissioning system 1300 < *Z*^2^*A*^−1/3^ < 1550 is tracking the super-long fission trends, TKE_*SL*_ = 0.1542*Z*^2^*A*^−1/3^ − 48.7 MeV.

We could very well see that the slope of TKE_*SL*_ predicts the TKE_sym_ of smaller fissioning system such as ^227^Ac but not for indefinitely smaller fissioning system. TKE_sym_ given by Brosa^17^ for ^213^At and ^227^Ac are 146 MeV and 153 MeV respectively, and TKE_sym_ predicted by TKE _*SL*_ are 137.9 MeV and 151.5 MeV for each fissioning system. Thus for fissioning system that are decreasingly smaller, the steep slope of TKE_*SL*_ might taper slightly. Systematics from calculated TKE_sym_ for fissioning system *Z*^2^*A*^−1/3^ > 1550 effectively gives the trend for super-short fission modes, TKE_*SS*_ = 0.0849*Z*^2^*A*^−1/3^ + 99.0 MeV shows that the super-short TKE is much flatter. The prediction of the TKE_asy_ and TKE_sym_ are quite excellent but there are too much asymmetric fragments in the calculation. This could probably be fixed by adopting the more realistic microscopic transport coefficients.

### The Trajectories

In order to explain why our calculations are able to reproduce the correlations between mass- and TKE-distributions, the immediate idea would be to analyze the potential energy *U*(*q*) for 3D and 4D calculations. This turns out to be very complicated due to the large dimensions involved. Neither does minimizing *U*(*q*) with respect to *δ* in every (*z*_0_/*R*_0_, *α*) coordinates gives any useful information because it cannot discriminate between forbidden and allowed fission paths, especially for the heavier actinides. It makes some sense to look at how *δ* is distributed at scission because the failure of minimization in *δ* indicates that the fission paths in *δ*-space might be crucial for the shape of the fission yield.

**Figure 5:**
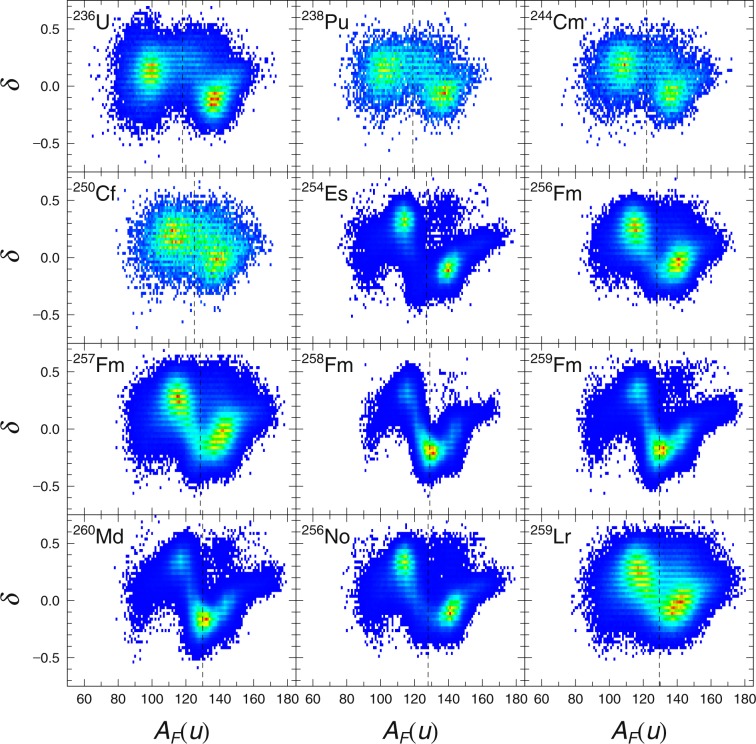
Distributions of *δ* as a function of fragment mass, *A*_*F*_(*u*). Dashed line indicate the symmetric fission fragment mass. The colors of the distribution scales linearly from blue to red indicating the increasing counts in arbitrary units.

Thus we first look at the distribution of *δ* with respect to the fission fragment mass. Positive *δ* means that the fragment tip is prolate, negative *δ* means that it is oblate and *δ* = 0 imply that fragments tips are spherical. Comparing Figs 1 and 5 we can see that the standard fission modes correspond to positive *δ* for *A*_*L*_ and negative *δ* for *A*_*H*_. Super long fission modes have positive *δ* for both fragments. Super short fission modes have negative *δ* for both fragments. In Fig. 5, we see the dominant super short fission modes in the expected single-peak yield nuclei and all double peak yield nuclei has dominant standard fission modes.

**Figure 6:**
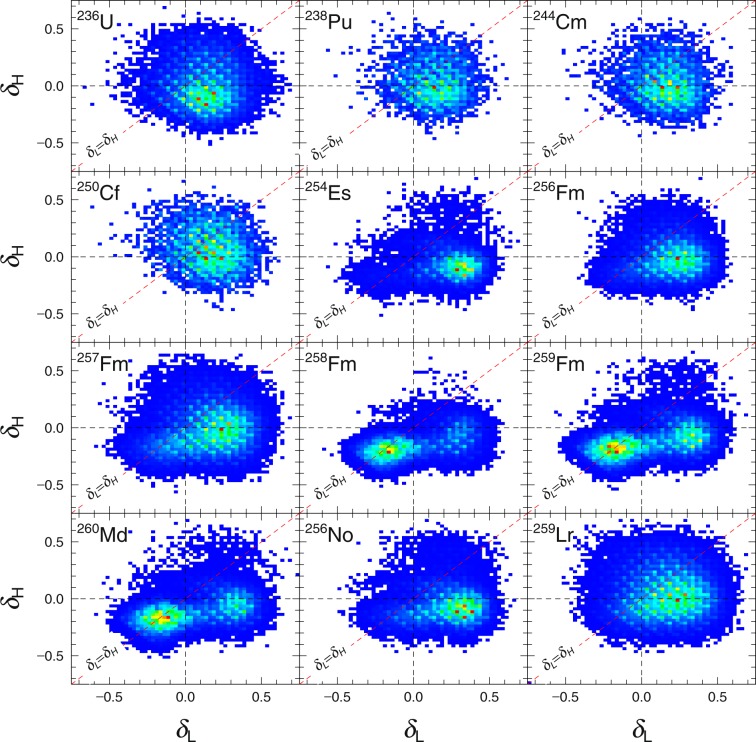
Combinations of *δ*_*L*_ and *δ*_*H*_ at scission for selected nuclei from ^236^U to ^259^Lr, summed over all possible asymmetries of fission fragments. Black dashed line indicate *δ*_*L*_ = 0 in x-axis and *δ*_*H*_ = 0 in y-axis. Red dashed line indicate *δ*_*L*_ = *δ*_*H*_. The colors of the distribution scales linearly from blue to red indicating the increasing counts in arbitrary units.

With that established, we can probably deduce what happened in the *δ*-space of *U*(*q*) by plotting the combination of *δ* for the light and heavy fission fragment denoted each by *δ*_*L*_ and *δ*_*H*_. Figure 6 immediately showed us the differences in the (*δ*_*L*_, *δ*_*H*_) combination for fissioning system smaller than ^254^Es against the ones equal to and larger than ^254^Es. The somewhat symmetrically distributed (*δ*_*L*_, *δ*_*H*_) combinations of ^236^U to ^250^Cf meant that in *δ*-space there is only a single fission path or at least the fission path are very close to each other. This fission path seem to lead to standard fission modes. The super-long fission mode fission path might be present but it seems to be very close to the fission path leading to standard fission modes. It also explain the success of 3-D Langevin model in describing them; after all a single fission path in *δ* meant that it was simply unnecessary to go to higher dimension. However, from ^254^Es (*δ*_*L*_, *δ*_*H*_) combinations became asymmetric. In ^258^Fm, ^259^Fm, ^260^Md it is revealed that the asymmetry of (*δ*_*L*_, *δ*_*H*_) are due to the presence of two fission paths in *δ*-space. The first fission path leads to the usual standard fission modes. The second one leads to the super-short fission modes. Due to the multiple fission path and asymmetry of (*δ*_*L*_, *δ*_*H*_) combinations, 3-D Langevin equations are unable to solve the transition between double-peak fission fragment yield to single peak fission yield. Now, with our 4-D Langevin, this is solved.

We can examine these conjectures regarding the relationship of the fission modes with the combinations of *δ*_*L*_ and *δ*_*H*_ by extracting the fission fragment yield for the specific case of *δ*_*L*_ ≈ *δ*_*H*_. All fission events satisfying the condition $|{\delta }_{1}-{\delta }_{2}|\le 0.025$ are collected and then we proceed to calculate the fission fragment yield associated with it. We may consider such procedure an approximation of 3-D Langevin calculations using 4-D Langevin equation. The approximated 3-D Langevin fission yield may then be compared with the fission yield calculated with 4-D Langevin fission yield that we had gave in Fig. 2.

**Figure 7:**
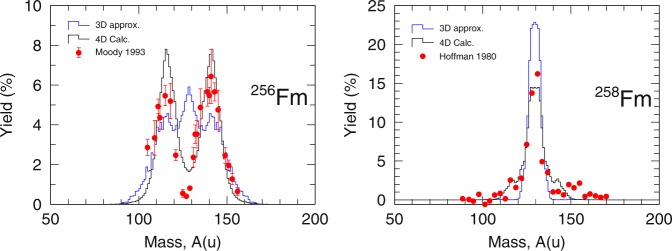
4-D Langevin calculated fission yield () and the approximation to 3-D Langevin () for ^256^Fm on the left and ^258^Fm on the right. Comparison with experimental data () for ^256^Fm^22^ and ^258^Fm^14^ are made for the respective figure from left to right.

In Fig. 7, we compare the approximated 3-D Langevin fission yield with the 4-D Langevin fission fragment yield for ^256,258^Fm. Our approximated 3-D Langevin fission yield displayed a three peak structure for ^256^Fm. The symmetric peak should originate from the super-short fission modes meanwhile the asymmetric peak should originate from the standard fission modes. If we reflect again to the combinations of *δ*_*L*_ and *δ*_*H*_ that we see in Fig. 6, we can see most the events associated with the standard fission modes for ^256^Fm is far off from the line for *δ*_*L*_ = *δ*_*H*_. Hence the dominance of the symmetric yield in approximated 3-D Langevin fission yield. In 4-D Langevin calculation of ^256^Fm, the super-short fission events are overwhelmed by the statistics from the standard fission events. Thus the fission yield by 4-D Langevin gave the familiar asymmetric double peak fission yield as was indicated from the experimental fission yield from Moody *et al*.^22^. The experimental fission yield gave an excellent fit of the calculated heavy fragment yield but the valley of the experimental fission yield is much lower than the valley of the calculated fission yield. The lighter part of the experimental fission yield are also slightly shifted to the lighter mass and the peak height lower. The slight misalignment of the calculated 4-D Langevin fission yield and experimental fission yield are most likely due to the prompt neutron emission in the experiment.

In the case of ^258^Fm, the approximated 3-D Langevin give the correct yield consistent with 4-D Langevin fission yield and experimental fission yield^14^. One deficiencies that we can see from the approximated 3-D Langevin is the thinness of the yield due to the lack of events with standard fission modes when *δ*_*L*_ = *δ*_*H*_. Apart from the slight misalignment of the peak for the calculated 4-D Langevin fission yield and experimental fission yield by Hoffman *et al*.^14^ most likely due to neutron emission, we can say that the experimental fission yield is well reproduced. Thus we now know the origins of the poor transition from ^256^Fm to ^258^Fm by 3-D Langevin and also perhaps of other methods relying on 3-D potential energy surface.

## The Model

**Figure 8:**
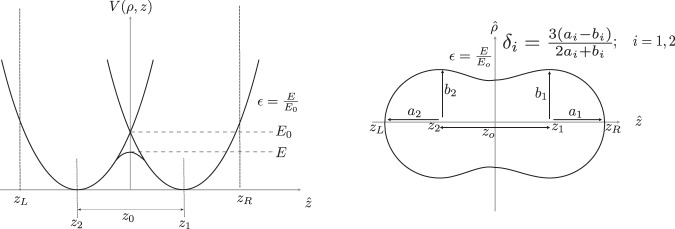
Left: Two centre shell model (TCSM) potential^37^. Right: Shape parameterizations based off the two centre shell model.

The 4-D Langevin approach describes the time evolution of the shape of nuclear surface defined by the TCSM collective variables, *q*_*μ*_ = (*z*_0_/*R*_0_, *δ*_1_, *δ*_2_, *α*). These collective variables are depicted in Fig. 8. There *z*_0_/*R*_0_ refers to the distance between the potential minimum of the left and right fragments, where ${R}_{0}=1.2\sqrt[3]{A}$ is the radius of spherical nucleus with mass number *A*. The parameters *δ*_*i*_ = 3(*a*_*i*_ − *b*_*i*_)/(2*a*_*i*_ + *b*_*i*_), where i = {1, 2}, describe the deformation of the right and left fragment tips. The fourth parameter *q*_4_ is the mass asymmetry *α* = (*A*_1_ − *A*_2_)/(*A*_1_ + *A*_2_) and it depends on the volumes of system to the left and right from the point *z* = 0. The fifth parameter of TCSM shape parameterization *ε*, defined as the ratio of actual and oscillator potentials at *z* = 0, *ε* ≡ *E*/*E*_0_, see Fig. 8, was kept constant, *ε* = 0.35, in all our calculations.

The time dependence of collective variables *q*_*μ*_ and the conjugated momenta *p*_*µ*_ is described by the system of first order differential equations (Langevin equations),

1 $$\begin{array}{rcl}\frac{d{q}_{\mu }}{dt} & = & {({m}^{-1})}_{\mu \nu }{p}_{\nu },\\ \frac{d{p}_{\mu }}{dt} & = & -\frac{\partial U(q)}{\partial {q}_{\mu }}-\frac{1}{2}\frac{\partial {m}_{\nu \sigma }^{-1}}{\partial {q}_{\mu }}{p}_{\nu }{p}_{\sigma }-{\gamma }_{\mu \nu }{m}_{\nu \sigma }^{-1}{p}_{\sigma }+{g}_{\mu \nu }{R}_{\nu }(t),\end{array}$$

where the sums over the repeated indices are assumed. In Eq. (1) *U*(*q*) is the potential energy, the *γ*_*μν*_ and ${(m)}_{\mu \nu }^{-1}$ are the friction and inverse mass tensors and *g*_*μν*_*R*_*ν*_ is the random force.

The potential energy *U*(*q*) is calculated as the sum of liquid drop deformation energy^23^ and shell corrections^24,25^. The single particle energies are calculated with the deformed Woods-Saxon potential^26^ fitted to the aforementioned TCSM shape parameterizations.The collective inertia tensor *m*_*μν*_ is calculated based on the Werner-Wheeler approximation of the liquid drop mass tensor^27^. The friction tensor *γ*_*μν*_ is calculated from the wall-window friction formulation^28–32^. The random force are calculated as the product white noise *R*_*ν*_ and random force strength *g*_*μν*_. More details are specified in our previous publications^18,19^.

A single event of Langevin calculation typically begins from the second minimum or in the vicinity of it. If it fails to achieve scission configuration after 10,000 fm/c, the calculation is terminated. Typically 500,000 events are done per nuclei but we occasionally increase the number of events if it was a critical calculation.

## Summary

By describing the fission process in terms of 4-D Langevin equations we have shown that the anomalously high TKE seen in ^258^Fm, ^259^Fm and ^260^Md is the inevitable results of splitting of nucleus into two almost double magic fragments that are symmetric as have been speculated by Hoffman *et al*.^14^. However, unlike ^236^U that demonstrate super-long fission modes for the symmetric splitting, these three nuclei of ^258^Fm, ^259^Fm, ^260^Md and other fissioning systems around them have super-short fission modes. The differences between super-long fission modes and the super-short fission modes shows itself in the Coulomb repulsion energy between the fission fragments that is stronger in the latter fission mode due to its shorter shape. Then a further investigation revealed that the super-short fission modes are present for all nuclei heavier than Einsteinium. Hence, the mystery on why the super-short fission modes are preferred in ^258^Fm, ^259^Fm, ^260^Md are solved. We even see the slow disappearance of super-long fission modes, prominent in ^236^U and hardly identifiable for ^250^Cf. The present limitation of the calculation is due to the use of macroscopic transport coefficients that causes a slight underprediction of 〈TKE〉 for ^258^Fm, ^259^Fm, ^260^Md. Nevertheless, our analysis of *δ* distribution indicates that the allowance for *δ* in our 4-D Langevin calculation showed us multiple fission paths dependent on the combinations of *δ*_*L*_ and *δ*_*H*_. This has allowed us to explain how these transitions happen in a consistent manner.

Most of the figures had been included for M.D.U. PhD thesis manuscript^33^ prior to the submission of this paper.

## Supplementary information

Tabulated Total Kinetic Energy (TKE) Data of Fissioning System.

## Data Availability

All data generated or analyzed during this study are included in this published article (and its Supplementary Information files).
