# Supplementary material for: Correlated transitions in TKE and mass distributions of fission fragments described by 4-D Langevin equation
Source: Sci Rep. 2019 Feb 6;9:1525. doi: 10.1038/s41598-018-37993-7 (PMC6365521; doi:10.1038/s41598-018-37993-7)
Supplement: Supplementary file 1 — Tabulated Total Kinetic Energy (TKE) Data of Fissioning System. [file 41598_2018_37993_MOESM1_ESM.pdf]

Supplementary Information for Article in ⟨ SREP-18-31029 ⟩

Correlated transitions in TKE and mass distributions of fission fragments described by 4-D Langevin equation

Mark D. Usang, Fedir A. Ivanyuk, Chikako Ishizuka and Satoshi Chiba.

Tabulated Total Kinetic Energy (TKE) Data of Fissioning System.

This supplementary information for our paper on *Correlated transitions in TKE and mass distributions of fission fragments described by 4-D Langevin equation* are the data used in plotting Fig. 3 and Fig. 4. The nuclide calculated here are further analyzed for the rest of the results.

Z	A	$Z^2A^{-1/3}$	E_x	TKE_{asy}	N_{asy}	TKE_{sym}	N_{sym}	$\langle TKE \rangle$
92	236	1369.63	20.0	174.99	90712	162.72	10610	173.57
93	234	1403.54	20.0	177.30	2334	167.81	525	175.24
93	235	1401.55	20.0	177.36	3438	166.70	680	175.22
93	236	1399.57	20.0	176.40	4466	167.01	812	175.21
93	237	1397.60	20.0	177.76	4788	167.03	658	176.30
93	238	1395.64	20.0	177.09	4432	166.02	494	175.70
93	239	1393.69	20.0	176.98	5222	166.72	540	175.56
93	240	1391.75	20.0	176.47	4990	165.54	496	175.45
94	236	1429.83	20.0	180.82	6360	172.17	1100	178.77
94	237	1427.81	20.0	180.84	6374	172.50	882	178.93
94	238	1425.81	20.0	180.27	8632	171.02	1160	178.41
95	239	1454.27	20.0	184.71	11034	174.27	1323	182.34
95	240	1452.25	20.0	184.26	9286	176.13	780	182.32
95	241	1450.24	20.0	183.42	9990	174.59	689	181.59
95	242	1448.24	20.0	182.72	7978	174.37	598	181.65
95	243	1446.25	20.0	183.06	7958	175.09	455	181.40
96	243	1476.86	20.0	186.36	13362	177.83	1969	185.18
96	244	1474.84	20.0	186.46	13962	179.45	1974	184.80
96	245	1472.83	20.0	185.60	9554	179.00	1347	184.16
97	244	1505.72	20.0	189.79	10768	183.81	1062	188.04
97	245	1503.67	20.0	189.54	10618	182.37	950	187.84
97	246	1501.63	20.0	189.49	8330	182.18	756	187.64
97	247	1499.60	20.0	188.51	8440	182.97	495	187.29
98	245	1534.84	20.0	193.08	18712	187.87	2565	191.47
98	246	1532.75	20.0	192.22	13868	186.65	1850	190.83
98	247	1530.68	20.0	191.92	10360	186.88	1293	190.68
98	248	1528.62	20.0	192.16	10490	188.10	1338	190.26
98	249	1526.57	20.0	191.69	7288	186.62	941	190.26
98	250	1524.53	20.0	189.88	8978	187.78	1602	189.88
99	254	1547.60	8.0	196.52	269138	229.11	4910	197.39
100	256	1574.90	7.2	196.63	378838	226.63	46014	200.01
100	257	1572.85	7.0	197.59	407186	232.71	67130	202.55
100	258	1570.82	7.5	198.48	101214	234.48	298650	226.17
100	259	1568.79	8.0	196.19	218742	234.15	415066	221.06
101	260	1598.27	8.0	199.07	188972	238.54	454580	226.69

102	256	1638.52	7.0	205.46	256684	235.26	37180	208.95
102	258	1634.28	7.0	204.83	335112	240.04	105840	213.43
103	259	1664.33	7.0	208.17	341838	240.57	45972	211.13
104	258	1699.00	7.0	213.54	251500	240.98	27298	217.17
104	260	1694.63	7.0	212.68	312390	244.44	77920	220.20

In this table, the variable Z, A and E_x denotes respectively the charge, mass and excitation energy associated with the nuclide in question. The fissioning system $Z^2A^{-1/3}$ of the fissioning nucleus is related to the Coulomb repulsion of fission fragments. The quantities $\mathrm{TKE}_{\mathrm{asy}}$ and $\mathrm{TKE}_{\mathrm{sym}}$, are the TKE associated respectively with asymmetric and symmetric mass splits. The numbers of fission events ending up with the asymmetric and symmetric mass splits in our calculations are given by N_{asy} and N_{sym} respectively from which we determine the dominant fission modes. The average TKE, $\langle \mathrm{TKE} \rangle$ is calculated by taking the sum of the TKE for each fission event and dividing it with the total number of fission events.

The calculations for the isotopes with Z number between 92 to 98 are done with $E_x = 20$ MeV. The fission calculations with Langevin equation at such low excitation energy are quite common and they are equivalent to the excitation energy of fission events by the incident 14 MeV neutrons. At higher Z number, the neutron induced fission data are quite scarce but spontaneous fission data are quite abundant. The initial excitation energy in spontaneous fission should be close to zero but the fission calculations with Langevin equation with zero excitation is impossible. The system would never cross the fission barrier. Thus, we start our calculation with the lowest excitation energy. One can see from the table that for Z number beyond 98, the excitation energy of most isotopes is between 7 and 8 MeV.
